# Quality Assessment in Paediatric Cardiology: Experiences from Leveraging a Clinical Data Warehouse

**DOI:** 10.3390/life16060941

**Published:** 2026-06-02

**Authors:** Wolfgang Wällisch, Sven Dittrich, Ariawan Purbojo, Isabelle Schöffl, Thomas Ganslandt, Hans-Ulrich Prokosch, Lorenz A. Kapsner, Jonathan M. Mang

**Affiliations:** 1 Department of Paediatric Cardiology, Universitätsklinikum Erlangen, Friedrich-Alexander-Universität Erlangen-Nürnberg (FAU), 91054 Erlangen, Germany; 2 Department of Paediatric Cardiac Surgery, Universitätsklinikum Erlangen, Friedrich-Alexander-Universität Erlangen-Nürnberg (FAU), 91054 Erlangen, Germany; 3 Medical Informatics, Friedrich-Alexander-Universität Erlangen-Nürnberg (FAU), 91058 Erlangen, Germany; 4 Institute of Radiology, Universitätsklinikum Erlangen, Friedrich-Alexander-Universität Erlangen-Nürnberg (FAU), 91054 Erlangen, Germany; 5 Medical Center for Information and Communication Technology, Universitätsklinikum Erlangen, 91054 Erlangen, Germany

**Keywords:** congenital heart defects (CHDs), risk stratification, mortality, data processing, real-time analytics, quality metrics

## Abstract

Background: The generation of quality metrics for paediatric heart centre programmes frequently relies on registry data, with all the known benefits and disadvantages. This retrospective monocentric study introduces an algorithm capable of processing unedited clinical data to identify mortality risk factors following paediatric cardiac surgery. Methods: Patients who had undergone cardiac surgery in the department during the period from 2011 to 2020 were included when aged < 18 years. Congenital heart disease (CHD) was categorised into four diagnosis groups through hierarchical integration of the index surgery and CHD diagnosis. We evaluated preoperative, demographic, periprocedural, and postsurgical risk factors. Results: A total of 1700 patients with 2157 hospitalization encounters were included. The risk factors for elevated mortality with the highest degree of significance were extracorporeal membrane oxygenation (hazard ratio 13.97, *p* < 0.001), weight < 2500 g, patients in the univentricular heart group I, and the creatinine ratio. Conclusions: Beyond confirming established predictors such as ECMO and low body weight < 2500 g, the present analysis highlights the creatinine ratio as a strong laboratory-based predictor of mortality. The applied framework serves as a foundational step towards enabling the real-time utilisation of raw datasets across multiple centres, thereby supporting privacy-preserving and efficient quality metric assessment as well as enhanced risk stratification.

## 1. Introduction

Congenital heart defects (CHDs) are a major cause of congenital malformations and still the leading cause of mortality from birth defects, accounting for more than one-fifth of all deaths [1]. The overall global prevalence of CHD has increased substantially over the past two decades (by 28%), particularly driven by more accurate preoperative diagnostics [2] and rising numbers of surviving adolescents and adults with congenital heart disease, resulting in a global prevalence around 158 per 100,000 people [3].

Recognition of modifiable risk factors for survival is crucial for improved clinical outcome, and measuring mortality and morbidity is an important feature of quality assessment. Due to the diverse spectrum of congenital heart disease, establishing formal risk stratification is demanding. Certain risk factors such as lower weight, preterm birth, and more complex CHD have been implemented in predicting mortality [4,5,6,7]. Multicentre consortia, real-world databases, networks, and voluntary registries such as the Society for Thoracic Surgery (STS), European Association for Cardio-Thoracic Surgery, and the German Registry for Cardiac Operations and Interventions in Patients with Congenital Heart Disease have been established with the intention to facilitate research at a larger scale in the area of quality of care [8]. Clinical registries have been shown to capture useful and important data.

However, in Central Europe, and particularly in Germany, the present decentralised hospital structure and General Data Protection Regulation (GDPR) in combination with national laws still constitute a major hurdle for comparing outcomes across multicentre approaches outside registry networks. The employment of various data collecting systems in each hospital makes it even more time consuming and sometimes practically impossible to gather and summarize information in a timely fashion.

MIRACUM (Medical Informatics in Research and Care in University Medicine) is a consortium established in 2018 as part of the German Medical Informatics Initiative (MII), a programme that is funded by the German Federal Ministry of Education and Research (BMBF) [9]. The primary objective of the initiative is to establish a nationwide infrastructure for the utilisation and sharing of health care data, with the ultimate goal of improving the quality of medical care and facilitating research [10]. Using the MIRCAUM-supported infrastructure established at our university hospital, we developed and implemented an algorithm designed to systematically process clinical outcome and quality assessment data for paediatric cardiovascular patients treated surgically at our institution.

## 2. Materials and Methods

### 2.1. Study Cohort

This monocentric study was approved by the ethics committee of the Friedrich-Alexander University of Erlangen–Nuremberg (Ethic board number 21-297-Br), waiving the need for informed consent. The authors declare that this research was performed in compliance with the World Medical Association Declaration of Helsinki on Ethical Principles for Medical Research Involving Human Subjects. The study comprised a retrospective analysis of all patients < 18 years who underwent congenital cardiac surgery at the Paediatric Heart Centre of the University Hospital Erlangen (UHE) between 2011 and 2020. All patients with acquired heart disease, including children undergoing heart transplantation, were excluded. Due to different and unique comorbidities, we also ruled out premature babies < 37 weeks with persistent ductus arteriosus ligation. This is in line with other mortality and morbidity studies [11,12].

### 2.2. Cardiac Disease Classification

CHD diagnoses were based on the International Classification of Diseases, 10th Revision (ICD-GM, Q20–Q28). Surgical procedures were identified using the German Procedure Classification OPS, specifically chapter 5–35 (“Operations on cardiac valves, septa, and vessels adjacent to the heart”), an adapted version of the International Classification of Procedures in Medicine (ICPM). The OPS coding system can be viewed on the website of the German Federal Institute of Drugs and Medical Devices (BfArM-Classifications). The procedure codes (OPS codes) that define eligible surgical corrections of congenital heart disease are provided in Supplementary Materials Table S1.

We classified patients by combining the index surgery (code) and CHD diagnosis using a hierarchical approach. The fundamental component of risk stratification, and consequently the differentiation between the disease categories, was predicated on the respective in-hospital mortality risk estimate. We have therefore organised the complexity of the operation or combination of operations from highest to lowest according to Kumar et al. [13]. For instance, a patient who has undergone surgical correction of atrioventricular septal defect (AVSD) was classified as having simple biventricular heart disease on the basis of the low in-hospital mortality rate of 2.5%. By contrast, AVSD with TOF was classified as having complex biventricular disease. To account for certain combinations of diagnoses and procedures, we used both surgical and cardiac (ICD) codes to assign to a cardiac disease group [14]. In addition, when a procedure consisted of several different components, the assignment was also based on the procedure with the highest estimated mortality risk, not on the lifetime prognosis of the underlying heart defects [15]. Using this approach, cases were allocated to one of four distinct disease groups, namely univentricular heart disease 1 (UVH I), univentricular heart disease 2 (UVH II), biventricular simple heart disease (BV-S), and biventricular complex heart disease (BV-C). Supplementary Materials Table S1 lists the specific combinations of procedure and diagnosis codes (ICD) used for classification.

To guarantee a sufficient sample size for the analysis and to take into account the considerably disparate risk profiles of the palliation stages [16], the group UVH I comprised two distinct subcohorts of patients (UVH Ia and Ib). The first subcohort included patients undergoing the Norwood procedure, either as a primary procedure or as part of a comprehensive stage II surgery. The second subcohort included single-ventricle patients who underwent PA-banding or shunt placement as palliative surgery stage I. The UVH II group represented patients either undergoing stage II (bidirectional cavopulmonary connection [BCPC]) or stage III (total cavopulmonary connection [TCPC]) of the univentricular pathway palliation. The latter category was further subdivided into two subgroups (IIa and IIb) in order to address cases involving index surgery other than Glenn or Fontan procedures. The following schema provides a synopsis of the classification “univentricular heart disease”.

I. Stage I palliation (UVH group I)
  (a) Ia: Greater complexity level of initial palliation
    (Initial Norwood procedure)
  (b) Ib: Lower complexity level of initial palliation
    (Palliation with shunt or PA-banding)
II. Stage II or III palliation (UVH group II)
  (a) IIa: Second and third stage of single ventricle palliation
    (BCPC or TCPC)
  (b) IIb: Existing BCPC or TCPC plus index surgery other than the Fontan or Glenn procedure

In instances where the information concerning the diagnosis or procedure was deemed inconclusive, a review of the patients’ medical records was conducted.

### 2.3. Outcome and Risk Factor Variables

The primary outcome metric was hospital mortality. The risk factors were selected on the basis of an established association with mortality in the context of cardiac surgery and by clinical relevance [4,5,6,7]. These factors were then grouped into three categories: preoperative, perioperative, and postoperative elements. The potential sources of variation in the data include patient-related factors, such as age, weight, and sex; surgical-related factors, including aortic cross-clamp and bypass time, as well as open thorax; and disease-related factors, including associated syndromes and concomitant malformations. In the majority of cases, the presence of disease-related variables and postoperative complications, including infection, unplanned surgery, dialysis, and extracorporeal membrane oxygenation (ECMO), were identified using the relevant ICD or OPS codes. This approach was also selected for the variable “infection” to ensure that only clinically confirmed and coded infections were included in the analysis. Furthermore, laboratory parameters were analysed both preoperatively and within the first 72 h after surgery. To account for interindividual baseline differences, postoperative changes were expressed as ratios relative to the corresponding preoperative values when deemed clinically appropriate.

### 2.4. Imputation of Missing Values

Missing values of the dataset were imputed in instances where no more than 20% of values were missing. The R package missForest [17] was utilized for imputation, employing non-parametric missing value imputation for mixed-type data, resulting in an NRMSE of less than 0.01% and an PFC of 0.39%. According to Stekhoven and Buehlmann (2012), for both the NRMSE and the PFC, values close to 0 are indicative of good imputation. The iterative imputation method is based on a random forest. By averaging over multiple unpruned classification or regression trees, random forest inherently implements a multiple imputation scheme [17]. Features such as “days deceased after surgery”, which correctly contained missing values, were not imputed. However, these features were included in the dataset to determine the missing values of the remaining features.

### 2.5. Data Analysis

The harmonisation and analysis of the data were undertaken by the Data Integration Centre (DIZ) of UHE. The data elements were extracted from the clinical data warehouse (cDWH). This is referred to as a single source of truth. It is imperative to translate, standardise, and harmonise the various data elements relevant to our analysis, namely, patient-related, surgery-related, disease-related, and ICU-related information, which are stored in separate source systems (i.e., SAP^®^, QIMS^®^, ICM^®^) in the first place, to ensure accurate and efficient utilisation of the data. This capability can be further enhanced through the application of specific extraction scripts that leverage the data sharing standard HL7^®^FHIR^®^ (Fast Healthcare Interoperability Resources). The MII employs this instrument to guarantee nationwide and interconsortial interoperability, thereby facilitating real-time data utilisation and transfer across hospitals and federal states in Germany. Since some data elements (such as operation details) were not yet modelled in the MII core dataset modules, these data elements were extracted from the clinical data warehouse (cDWH) for this work. To ensure that the same laboratory examinations could be correctly recognized and compared across the different laboratories, the laboratory-specific examination/analyte codes were mapped to LOINC. Examinations with the same LOINC code were pooled across the different laboratories and subsequently analysed collectively. The mapping of the local analyte codes to the LOINC terminology was performed by an expert and subsequently validated by the participating laboratories.

### 2.6. Statistical Analysis

The Cox proportional hazard (CPH) regression model [18] is a well-established and widely applied method for survival analysis [19,20].

In this analysis, CPH models were fitted to evaluate the association of selected risk factors with the primary outcome in-hospital mortality. Hazard ratios (HR) with 95% confidence intervals were reported as effect measures. To account for the fact that multiple hospitalizations per patient were available in the dataset, a Cox regression with mixed effects (frailty model) was employed. This approach introduces random effects to account for within-patient correlation, thereby avoiding bias from the violation of the CPH’s assumption of independence inherent in conventional regression models [21].

In addition to survival analysis, logistic regression models were applied for binary outcomes. Odds ratios (OR) with 95% confidence intervals were calculated to quantify the strength of associations between covariates and dichotomous endpoints. *p*-values in Table 1 are exploratory and descriptive. Potential multicollinearity among clinically interrelated variables was assessed prior to final model fitting by analysing correlation structures using the corrplot package in R. Due to strong correlations between patient weight and age at surgery, body weight < 2500 g was included as a binary variable, whereas the continuous weight variable was excluded. Similarly, height was removed because of substantial collinearity with age, and age at surgery was prioritised over age at hospital admission. For postoperative laboratory parameters obtained within the first 72 h after surgery, clinically relevant extreme values (maximum or minimum levels, depending on the parameter) were retained for analysis. Correlation structures among all remaining features were inspected to assess potential multicollinearity, using an absolute Pearson’s correlation coefficient threshold of 0.7.

Statistical analysis was performed in R, Version 4.5.1 using the following packages: arsenal, billboarder, corrplot, crosstable, data.table, DescrTab2, finalfit, flextable, Hmisc, mice, missForest, sjPlot, sjtable2df, stats, survival, and survminer.

## 3. Results

### 3.1. Cohort Description

The present study comprehended a total of 1700 patients who had 2157 documented hospitalization encounters. The distribution of heart disease groups is as follows: simple biventricular with 1240 cases (57.5%), complex biventricular with 530 cases (24.6%), univentricular group I with 163 cases (7.5%), and univentricular group II with 224 cases (10.4%). The UVHD I group is further subdivided into groups Ia with 98 cases and greater complexity level of initial palliation (4.5%) and Ib with 65 cases and lower complexity level of initial palliation (3.0%). It is evident that the data cohort encompasses the entire spectrum of paediatric congenital heart disease, with a significant proportion of complex cases (illustrated by the groups complex biventricular and UVH I). The majority of patients were neonates and infants, with 882 infants (40.8%) representing the largest group. Subsequent to this, neonates accounted for 20.9% of all cases (451). In both the univentricular group I and the complex biventricular group, neonates constituted the predominant portion of patients, with percentages of 73.6% and 43.2%, respectively. As would be anticipated, the median age at first operation was found to be lower in the more complex heart disease groups, with 7.4 and 9.4 days recorded in the UVH groups Ia and Ib, respectively. This was followed by the complex biventricular patient group, where 62 days (95% CI [8.4; 343.4]) were recorded. If the very few hospitalizations of children aged > 5 years are excluded, the median age in this group is even lower at 33 days.

As illustrated by the findings concerning median age, the median weight in these cohort groups was considerably lower, at 3.4 and 4.2 kg in the UVH I and complicated biventricular groups, respectively, in comparison to 6.3 kg in the uncomplicated biventricular group. The median bypass time for the entire study cohort was 121 min. As cardiac diseases become increasingly severe, the median bypass time has been shown to rise significantly in more complex surgical groups. The highest median bypass times were observed in the Norwood palliation group Ia, followed by the complex biventricular group (228.5 and 162.0 min, respectively). This correlation is also evident in the variables “days ventilated” and “days in intensive care”. The UVH group I and the complex biventricular group demonstrated the longest duration of stay and ventilation time.

Chromosomal abnormalities and concomitant extracardiac malformations were observed in approximately 13.1% and 13.3% of cases, respectively, falling within the expected range [12]. Of particular note was the finding that the proportion of concomitant malformations in UVD group Ib was notably high, with more than one-third of all cases showing this feature, and this was a significant increase compared to that found for UVD group Ib.

The cohort description indicated a relatively high number of cases of extracorporeal membrane oxygenation (ECMO) and dialysis in UVD groups I and II which exceeded the expected range. The complication rate was 27% in the UVH I group and 8% in the UVH II group for the ECMO procedure. For dialysis requirement, the rate was 26.4% in the UVH I group and 6.3% in the UVH II group. The distribution of further postoperative morbidities for the entire cohort, including infection (13.9%), open thoracotomy (8.4%), and unplanned operation (3.4%), was found to be within the usual scope [15]. The comprehensive demographic and pre-, intra- and postprocedural data are shown in Table 1.

### 3.2. Primary Outcome Mortality and Dependent Risk Factors

In the context of the comprehensive study cohort, which encompassed a total of 2157 index surgeries, a mortality rate of 3.6% was observed, with 77 deaths occurring during the patient’s hospital stay.

As anticipated, the mortality rate exhibited a marked increase in direct proportion to the complexity of the heart disease category, with seven deaths (0.67%) recorded in cases of simple biventricular disease, 28 deaths (5.3%) observed in instances of complex biventricular disease, and 19 deaths (19.4%) occurring within the UVH group Ia with Norwood palliation category. In patients with univentricular hearts who have undergone surgical palliation with shunt placement or PA banding (group Ib), a higher than expected mortality rate was observed [22], with 12 deaths (18.5%) recorded. The UVH group II showed a lower mortality with eight deaths.

The selection of candidate risk factors for mortality was conducted on the basis of empirical evidence and in accordance with the current literature. The following variables were collected: preoperative data, primarily patient-related elements, intraoperative data, and postprocedural factors. The bivariable associations between potential risk factors and mortality are outlined in Supplementary Materials Table S2.

There is a robust correlation between specific categories of heart disease and in-hospital mortality (*p* = 0.05). In addition to the escalating severity of the cardiac diagnosis group, the mortality rate exhibited an upward trend in correlation with decreasing age, lower weight, and increasing bypass time. It was striking that a significant proportion of the diseased patients (33.8%) were newborns in their first week of life. Furthermore, this figure remained significantly higher in comparison to newborns in the second to fourth week of life (33.8% vs. 22.1%). This finding was particularly corroborated by the observation that a weight of less than 2500 g at the time of the index surgery was a significant contributor to increased mortality in this cohort. The issue of whether subsequent refinement is necessary with regard to weight was the focus of a comparative analysis between cases involving patients with weights of less than 2500 g and less than 3000 g. It can be concluded from the data demonstrated that the mortality rate remained unaffected by the threshold weight of 3000 g. Intraoperative parameters, including deep hypothermia (<28 °C) and open thorax, demonstrated a strong correlation with elevated mortality rates.

Postoperative indicators of renal function, including creatinine and urea levels, in addition to leukocyte counts of less than 4000/μL, have been linked to a higher risk of death. Univariate analysis indicated that the occurrence of morbidities, such as extracorporeal membrane oxygenation (ECMO), dialysis, or infection and complications, such as unplanned operations, could serve as a predictor for increased in-hospital mortality. The data demonstrated a clear correlation between extended periods of mechanical ventilation and prolonged ICU stay and an augmented risk of mortality. The mean duration of ventilation was found to be 16.4 days for deceased patients in comparison to a mean duration of 1 day for survivors. Similarly, the mean duration of ICU stay was found to be 28.8 days in the non-survivor group in comparison to a mean duration of 7.5 days for survivors.

Predictor selection and model construction followed a theory-driven hierarchical block-entry approach based on clinical expertise, established medical guidelines, and previously reported risk factors from the domain-specific literature. The predictors were entered into the model in the following order: (1) heart disease group and patient-related factors, such as age and weight at time of index surgery; (2) surgery-related characteristics; and (3) significant pre- and postoperative variables.

The selection of predictors was primarily informed by three key factors. First, the predictors were selected based on their established association with mortality in the context of cardiac surgery. Second, the predictors were selected based on their clinical relevance. Third, the predictors were selected based on the significance of their correlations with the dependent variable in our previous bivariate analysis.

The preliminary step 1 model demonstrated a highly significant correlation between UVH I and complex biventricular in descending order when compared to simple biventricular repair. In addition, it was established that patients with a weight of less than 2500 g were at a substantial elevated risk of mortality (HR 5.75; 95% CI 2.63–12.58; *p* < 0001). Of note, the patient’s age was not a statistically significant predictor (*p* = 0.223) in the initial modelling of the data.

As outlined in Table 2, the final multivariate model confirmed that low weight (<2500 g) and the postoperative requirement of ECMO were the most suitable predictors for mortality in this cohort.

**Figure 1:**
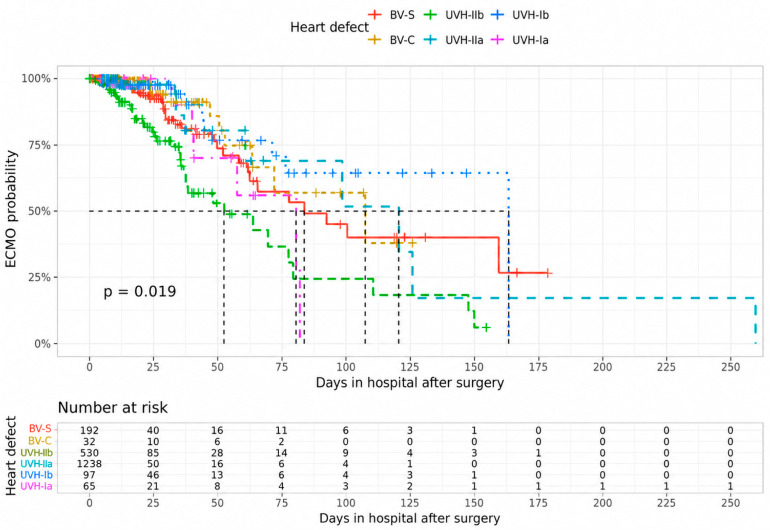
Kaplan–Meier estimates of time to ECMO support after cardiac surgery, stratified by congenital heart defect. Patients without an event were censored at hospital discharge. Tick marks denote censored cases. The table below the plot shows the number of patients at risk over time. Group comparisons were performed using the log-rank test (*p* = 0.019). Abbreviations: ECMO: extracorporeal membrane oxygenation; BV-S: biventricular. simple; BV-C: biventricular complex; UVH: univentricular heart disease.

The complication ECMO had by far the greatest negative impact on patient survival, with a 13.97-fold increase in the mortality rate in cases where ECMO was established at some point during the patient’s hospital stay, as illustrated in Figure 1.

It is evident that the presence of univentricular heart disease in the initial palliative stage (UVH group I) had strong impact on survival outcome. This is reflected in the data, which indicate that patients in group Ia had a higher probability of in-hospital death, with a calculated hazard ratio (HR) of 4.31. In a similar manner, patients in group Ib demonstrated a markedly elevated mortality rate, with a HR of 6.09 (95% CI 1.82–20.39; *p* = 0.01). To a lesser extent, but nevertheless with considerable significance, the creatinine ratio was found to be a contributing factor in explaining the observed mortality risk, with a 1.74-fold increase being recorded.

In the context of the present cohort, neither age at surgery (*p* = 0.137) nor the presence of certain morbidities, such as infection (*p* = 0.534) or unplanned operations (*p* = 0.730), was found to provide a more precise explanation model for mortality.

## 4. Discussion

### 4.1. Cohort Characteristics

The implementation of more accurate preoperative diagnostics, coupled with a consistent birth prevalence of congenital heart disease in high income countries [23], led to rising numbers of patients. With continuously evolving treatment strategies, the CHD related mortality rate has remained consistent and low during the past decade [4,5,6,7]. The overall mortality rate for all surgical procedures has been reported to range from 2.5% to 4.7% [4,5,6,7]. The in-hospital mortality rate was found to be 3.6% across the entire cohort in the present study. This must be viewed in the context of the synopsis of the cohort composition. As compared to other substantial mortality studies, the percentage share of complex patients with younger age was larger in this study, with approximately 7.5% of cases falling within the highest mortality risk category.

The predominant cardiac malformations observed in our cohort are those of simple heart defects, with more than half of the patients treated surgically falling into this category. The mortality rate was, as anticipated, low at 0.6% [24].

Substantial improvement in surgical outcomes has been realised worldwide, particularly in complex CHD [25]. The more aggressive and earlier approach to correct complex biventricular heart defects, as evidenced by the recent study of Erikksen et al. [14], has been adopted in our centre as well. This paradigm shift in surgical methodology entails a transition from palliative procedures to corrective surgery.

The strategy is reflected by the significant proportion of neonates in the complex biventricular group. Nearly half of this challenging cohort were neonates (43.2%), with 97 newborns (18.3%) treated in the first week of life. As illustrated by the groups complex biventricular and UVH I, these more complex patients accounted for approximately one-third of all cases. This was accompanied by a high proportion of neonates and infants in the entire cohort as well, with values of 20.9% and 40.8%, respectively. This is similar to or higher than the figures reported in many comparable mortality studies [4,5,6,7]. Age at surgery is known to be a risk factor for mortality and morbidity [26]. The median age at first operation was 62 days (95% CI [8.4; 343.4]) in the complex biventricular group and 7.4 days (6.4–29.9) in the UVH Ia group. In consideration of the aforementioned findings, the mortality rate in the group of complex biventricular cases was 5.3% in the present study. This result is in line with international data indicating a constant rate of less than 10% in these demanding patients over the last decade [1,27].

The classification of cases as belonging to the UVH group I category, which is characterised by univentricular heart disease in stage I palliation, has been demonstrated to exhibit the highest mortality rates at 18.5% (Ib) and 19.4% (Ia), respectively. The current literature reveals a clear correlation between the complexity of the heart disease, specifically the presence of hypoplastic left heart syndrome, and the reduced likelihood of survival [4,5,6,7]. It is noteworthy that a substantially elevated percentage of concomitant malformations (35.4% versus 13.3% in group Ia) were observed in the univentricular group Ib with shunt/conduit placement or PA banding as the primary palliative procedure. This finding may provide a rationale for the elevated mortality rate of 18.5% observed in the UVH Ib subgroup. In addition to the elevated incidence of malformations in the UVHD group Ib, chromosomal abnormalities and concomitant malformations demonstrated no substantial disparities among the various heart disease groups. The occurrence of these patient related factors in our study with 13.3 and 13.1%, respectively, was comparable to that reported in the current literature, with a prevalence ranging from 14 to 20% [12].

### 4.2. Primary Outcome Mortality and Risk Factors

Risk stratification is contingent on the presence of known risk factors, which, in an ideal scenario, are capable of being modified. The categorisation of the selected candidate risk factors for mortality in the present study was conducted in accordance with the following variables: preoperative, intraoperative, and postprocedural. This approach was adopted to consider the various impacts of underlying pathologies and the individual patient-specific factors [26].

A notable negative correlation between patient-related factors and mortality was identified, with younger age groups and lower weight representing particularly relevant factors in the bivariate analysis. These specific risk factors have been shown to be comparatively robust in terms of their association with mortality in the available literature [27,28,29]. In the study cohort, the presence of other preprocedural factors, including previous surgical admissions and the presence of chromosomal aberrations, did not prove to be of significant predictive value. This finding is in contrast to the results of a recently published study of the ECHSA database [30], in which the prevalence of non-cardiac disease (trisomy 21, 22q11 deletion) was identified as a significant factor contributing to operative mortality. One potential explanation for the observed discrepancy may be attributed to the relatively low incidence of cases with non-cardiac disease in this particular study, with a value of 4.6% compared to 13.1% in our own dataset. A substantial proportion (37.7%) of diseased patients exhibited concomitant malformations, a finding that was found to have a significant correlation with elevated mortality rates in the context of univariate analysis. Intra- and postoperative factors, such as open thorax and ECMO, respectively, are also factors contributing to increased mortality.

It is interesting to highlight that, in the context of the assessment of laboratory values, a clear relationship was demonstrated between kidney parameters, such as the creatinine ratio and the urea ratio, and adverse outcome. This relationship was also evident in cases with low leucocyte counts (<4000/µL), although this finding was limited to the bivariate analysis. However, no other inflammatory markers evaluated in the study yielded similar results. Despite the lack of independent significance in the multivariate model, low leucocyte counts remain of particular interest, as this variable had previously demonstrated relevant predictive importance in a machine learning–based mortality analysis conducted by our group [31].

In the multivariable analysis, the most accurate prediction model of mortality in the present cohort comprised a postoperative requirement of ECMO, a preprocedural weight of <2500 g, assignment to univentricular heart group I, and an increased creatinine ratio. The variable ECMO had the greatest negative impact on patient survival, increasing the mortality rate by 13.97-fold in cases where ECMO was established at some point during the patient’s hospital stay. The application of extracorporeal membrane oxygenation is acknowledged as a major risk factor for unfavourable outcomes [32], as confirmed by several studies and most recently by an analysis reported from the European Congenital Heart Surgeons Association (ECHSA) [30].

The patient-level factor that exerted the most significant influence across all models was a weight of less than 2500 g, which resulted in a mortality rate that was more than eight times higher when applicable. In the context of the entire cohort, the multivariate analysis revealed that other patient-level factors, including age, sex, and general weight, did not enhance the predictive capability of the outcome mortality model. Whilst the literature has largely corroborated the premise that a weight below 2500 g constitutes a risk factor [33], a heterogeneity of conclusions has been reached for the variables of age, specifically neonates, and weight and their prognostic value [4,5,6,7]. Nevertheless, despite the large percentage of neonates in our current dataset of surgical patients, inclusion of the variable “neonate” as a standalone factor did not enhance the predictive power in our model.

The initial procedural stage of the univentricular pathway is associated with a considerable mortality and morbidity risk [4,5,6,7]. This applies irrespective of whether the palliation involves a Norwood procedure, aortopulmonary shunts, or surgical banding of the pulmonary artery [34]. In the present analysis, the correlation between these procedures and a higher event rate was also demonstrated to be statistically significant. This observation was also made in relation to group UVH Ib, for which the predictive prowess for mortality estimation was found to be even higher (hazard ratio 6.09, *p* = 0.010). This finding indicates that these palliative surgical procedures are associated with a substantial risk, thereby emphasising the necessity for heightened awareness among the treatment team.

In the course of the ongoing refinement of mortality risk models, Jacobs et al. identified preoperative renal dysfunction as a significant driver of elevated mortality following paediatric cardiac surgery [26]. A meta-analysis with assessment of acute kidney injury in children undergoing cardiac surgery demonstrated higher rates of in-hospital mortality, renal replacement therapy, and cardiac arrhythmias in patients suffering from AKI [35,36]. The estimated mortality rate among children with AKI is around 9–15% [4,5,6,7], with substantially higher numbers up to 31% in the neonatal population [36].

The creatinine ratio, whilst not the most significant variable in terms of its importance for the mortality prediction model in our cohort, did demonstrate a noteworthy level of feature contribution. It was observed to exhibit enhanced predictive performance and found to be associated with a 1.74-fold elevated mortality risk. In another study of our working group in a similar cohort, we were also able to highlight the importance of renal function assessment using machine learning models and feature importance. Maximum serum creatinine (within 72 h postoperatively) was the top feature and strongest predictor of mortality in this analysis [31]. Given the large percentage (30–50%) of AKI in high risk populations [37] and the established link between cardiac surgery and neonatal AKI [4,5,6,7], it is important to mention that creatinine (and/or urea) levels were, in contrast to our findings, not a major contributing factor in the extensive database and mortality studies conducted by Bertsimas [34] and Du et al. [38]. This might partly be due to lower proportion of neonates and less complex cases. Furthermore, we know that most patients recover from kidney dysfunction after paediatric cardiac surgery within a short period of time [39]. Altogether, we think it is crucial to integrate a kidney function parameter into prediction models assessing outcomes after cardiac surgery.

Cardiac surgery is known to impose significant stress on various organ systems. The specific way in which the body responds to this physical demand is likely to vary considerably [6]. The emphasis on individual patient factors and their interactions has led to the development of more sophisticated prediction models of morbidity and mortality, capable of capturing more intricate and nuanced outcomes [4,5,6,7]. Over the past decade, certain factors have emerged as being robustly associated with a decreased survival rate. These include a worse preoperative status and conditions such as low weight (less than 2500 g) and complex heart disease [1]. The present datasets confirmed these factors, with the variables demonstrating the greatest prognostic value being the requirement for ECMO, a preprocedural weight of less than 2500 g, assignment to univentricular heart group I, and an increased creatinine ratio.

In light of an increasingly individualised approach, novel techniques are being employed to capture the big picture. It is imperative that the potential nonlinear interactions of risk factors are taken into account and addressed in prediction models [1,34,40]. Registries and databases (for example, the German Quality Assurance For Congenital Heart Diseases or STS National Database) and corresponding model prediction are dependent on given datasets with predefined data fields [41]. Consequently, it is possible that not all significant or newly detected variables and their interactions will be encompassed within the sample [6]. In contrast, network technologies, data model standards (e.g., HL7 FHIR^®^), and the corresponding data integration employed partly in the present analysis enable the homogenisation of unedited datasets across different centres and the addition of new variables in real time [10,42,43].

To further validate and refine this automated pipeline for real-time prognostic decision support, prospective multicentre studies are warranted. Future investigations should assess the framework’s predictive performance using established measures of discrimination and calibration, including the concordance index (C-index), and explore the incorporation of dynamic laboratory parameters, such as the creatinine ratio, as time-dependent covariates to more accurately capture the evolving clinical status of patients in the intensive care unit.

In recent years, novel AI analysis tools and methodologies have empowered researchers to manage large datasets effectively and enhance the efficacy of quality assessment and risk stratification [31,34,44]. The present study employed traditional logistic regression to confirm the comparability of the data with the available literature.

## 5. Limitations

It is important to consider these findings within the context of their limitations.

In the present work, analyses were conducted using data from a single centre. While this limits the generalisability of our findings, we are aware of the advantages of the German Medical Informatics Initiative (MII) in enabling multicentre, standardised research. Since not all relevant data were yet available in structured and harmonised form (via HL7 FHIR^®^), a broader integration was beyond the scope of this study but represents an important perspective for future work.

In light of the retrospective nature of the analysis, there is an inherent reliance on the availability and completeness of the data. The percentage of missing data related to the laboratory values is between 2 and 20%, which may therefore act as a confounder. Conversely, a reliable method has been utilised to impute these values accurately [17].

A limitation of the cardiology diagnosis groups is their reliance on ICD and OPS codes, which have been known to be misleading if not accurately coded. In order to ascertain the accuracy of the algorithm, a comparison was made between the dataset and two other registries, the German national registry and the ECHSA database, which regularly receive data on surgical and interventional procedures from the present centre. The findings demonstrated comparable case numbers and a proportion of complex operations that was consistent with the expected accuracy of the algorithm.

In addition, strategies employing the International Classification of Diseases (ICD) code approach have been successfully utilised in adult patients [45,46] and for paediatric AKI assessment as well [36]. The field of paediatric cardiology and congenital heart surgery is characterised by a broad spectrum of disease severity, diagnosis, and surgical procedures. This also applies to the present dataset, complicating categorisation processes and frequently failing to consider the interaction between surgical and patient factors. The severity of the condition can vary significantly even within the same category, making precise prognoses and risk stratification challenging [22]. Furthermore, the present study did not contribute to our understanding of the composition of, or the immediate causes of, death. This is a clear shortcoming of the study’s methodology.

## 6. Conclusions

Conventional databases are limited in their capture of relevant variables and their interactions, particularly in the context of newly emerging variables.

The proposed framework in the present study highlights the potential of automated analysis of original and unprocessed datasets to enable scalable, privacy-preserving, and near real-time risk stratification and quality assessment across paediatric cardiac centres.

The implemented model revealed that the most significant prognostic factors for mortality were found to be the need for ECMO, a preprocedural weight below 2500 g, univentricular heart group I classification, and an elevated creatinine ratio.

## Figures and Tables

**Table 1:** Cohort characterization across clinical encounters: demographic, procedural, and patient-level variables stratified by congenital heart defect groups.

Variable	Category		Congenital Heart Defect Groups					
		BV-S	BV-C	UVH-IIa	UVH-IIb	UVH-Ia	UVH-Ib	*p* Value
Deceased	False	1233 (99.4%)	502 (94.7%)	184 (95.8%)	29 (90.6%)	79 (80.6%)	53 (81.5%)	<0.001 ^1^
	True	7 (0.6%)	28 (5.3%)	8 (4.2%)	3 (9.4%)	19 (19.4%)	12 (18.5%)	
Sex	M	658 (53.1%)	324 (61.1%)	129 (67.2%)	23 (71.9%)	70 (71.4%)	36 (55.4%)	<0.001 ^2^
	W	582 (46.9%)	206 (38.9%)	63 (32.8%)	9 (28.1%)	28 (28.6%)	29 (44.6%)	
Age at surgery (days)	Med [IQR]	268.4 [127.4; 1494.4]	62.0 [8.4; 343.4]	311.4 [153; 1196]	1424.9 [717; 4639]	7.4 [6.4; 29.9]	9.4 [6.4; 31.4]	<0.001 ^3^
	Mean (std)	1134.4 (1659.7)	654.1 (1382.0)	716.9 (684.1)	2508.5 (2120.7)	67.8 (175.3)	27.8 (40.6)	
Age cohort	Neonates (0–28 d)	102 (8.2%)	229 (43.2%)	0 (0%)	0 (0%)	72 (73.5%)	48 (73.8%)	<0.001 ^2^
	Infants (29–365 d)	571 (46.0%)	171 (32.3%)	98 (51.0%)	3 (9.4%)	22 (22.4%)	17 (26.2%)	
	Small children (1–3 years)	252 (20.3%)	55 (10.4%)	70 (36.5%)	14 (43.8%)	4 (4.1%)	0 (0%)	
	≥4 years	315 (25.4%)	75 (14.2%)	24 (12.5%)	15 (46.9%)	0 (0%)	0 (0%)	
Weight (kg)	Med [IQR]	7.4 [5.0; 15.0]	4.2 [3.3; 8.2]	8.1 [6.2; 14.0]	15.0 [12.0; 34.2]	3.4 [3.1; 4.0]	3.4 [2.9; 3.7]	<0.001 ^3^
	Mean (std)	14.2 (16.6)	9.1 (12.3)	10.3 (5.1)	23.8 (17.2)	4.1 (2.1)	3.5 (1.0)	
Bypass time (min)	Med [IQR]	113.0 [61.0; 169.0]	162.0 [78.5; 227.0]	95.5 [68.0; 126.2]	116.5 [0; 155.0]	228.5 [188.5; 278.5]	58.0 [26.0; 112.0]	<0.001 ^3^
Bypass time group (min)	0 min	201 (16.2%)	70 (13.2%)	0 (0%)	10 (31.2%)	0 (0%)	15 (23.1%)	No test
	>0–<90 min	94 (7.6%)	34 (6.4%)	35 (18.2%)	0 (0%)	0 (0%)	19 (29.2%)	
	≥90 min	945 (76.2%)	426 (80.4%)	157 (81.8%)	22 (68.8%)	98 (100%)	31 (47.7%)	
Minimal temperature during surgery (group)	>32 °C	378 (30.5%)	133 (25.1%)	87 (45.3%)	12 (37.5%)	1 (1.0%)	40 (61.5%)	<0.001 ^2^
	28–32 °C	539 (43.5%)	109 (20.6%)	86 (44.8%)	10 (31.2%)	12 (12.2%)	18 (27.7%)	
	<28 °C	323 (26.0%)	288 (54.3%)	19 (9.9%)	10 (31.2%)	85 (86.7%)	7 (10.8%)	
Weight < 2500 g	False	1214 (97.9%)	498 (94.0%)	191 (99.5%)	32 (100.0%)	97 (99.0%)	60 (92.3%)	<0.001 ^1^
	True	26 (2.1%)	32 (6.0%)	1 (0.5%)	0 (0%)	1 (1.0%)	5 (7.7%)	
ECMO	False	1227 (99.0%)	494 (93.2%)	180 (93.8%)	26 (81.2%)	63 (64.3%)	56 (86.2%)	<0.001 ^1^
	True	13 (1.0%)	36 (6.8%)	12 (6.2%)	6 (18.8%)	35 (35.7%)	9 (13.8%)	
Days on ventilator	Med [IQR]	0.5 [0.2; 1.3]	3.1 [1.1; 7.9]	0.4 [0.2; 1.9]	2.1 [0.6; 21.9]	13.0 [6.2; 22.4]	6.2 [1.3; 19.1]	<0.001 ^3^
Days in ICU	Med [IQR]	2.0 [1.2; 3.8]	7.2 [3.0; 14.8]	2.0 [1.2; 3.1]	3.0 [2.1; 5.9]	15.5 [11.4; 31.5]	12.5 [8.8; 21.1]	<0.001 ^3^
Dialysis	False	1207 (97.3%)	484 (91.3%)	180 (93.8%)	30 (93.8%)	67(68.4%)	53 (81.5%)	<0.001^1^
	True	33 (2.7%)	46 (8.7%)	12 (6.2%)	2 (6.2%)	31(31.6%)	12 (18.5%)	
Chromosomal abnormalities	False	1023 (82.5%)	476 (89.8%)	188 (97.9%)	31 (96.9%)	96(98.0%)	61 (93.8%)	<0.001 ^1^
	True	217 (17.5%)	54 (10.2%)	4 (2.1%)	1 (3.1%)	2 (2.0%)	4 (6.2%)	
Infection	False	1135 (91.5%)	414 (78.1%)	172 (89.6%)	23 (71.9%)	69(70.4%)	45 (69.2%)	<0.001 ^1^
	True	105 (8.5%)	116 (21.9%)	20 (10.4%)	9 (28.1%)	29 (29.6%)	20 (30.8%)	
Unplanned operations	False	1221 (98.5%)	499 (94.2%)	184 (95.8%)	30 (93.8%)	91(92.9%)	59 (90.8%)	<0.001 ^1^
	True	19 (1.5%)	31 (5.8%)	8 (4.2%)	2 (6.2%)	7 (7.1%)	6 (9.2%)	
Number of unplanned operations	Med [IQR]	0 [0; 0]	0 [0; 0]	0 [0; 0]	0 [0; 0]	0 [0; 0]	0 [0; 0]	<0.001 ^3^
Creatinine change after surgery (%)	Med [IQR]	1.2 [1.0; 1.3]	1.3 [1.1; 1.5]	1.2 [1.1; 1.4]	1.2 [1.1; 1.3]	1.4 [1.2; 1.7]	1.3 [1.0; 1.6]	<0.001 ^3^
Urea change after surgery (%)	Med [IQR]	1.2 [0.9; 1.7]	1.7 [1.1; 2.6]	1.3 [1.0; 1.7]	1.1 [0.9; 1.7]	2.3 [1.6; 3.1]	1.5 [1.1; 2.2]	<0.001 ^3^
Leukocytes below 4000 after surgery	False	1212 (97.7%)	504 (95.1%)	187 (97.4%)	32 (100.0%)	79 (80.6%)	62 (95.4%)	<0.001 ^1^
	True	28 (2.3%)	26 (4.9%)	5 (2.6%)	0 (0%)	19 (19.4%)	3 (4.6%)	
Open thorax	False	1217 (98.1%)	466 (87.9%)	183 (95.3%)	27 (84.4%)	32 (32.7%)	50 (76.9%)	<0.001 ^1^
	True	23 (1.9%)	64 (12.1%)	9 (4.7%)	5 (15.6%)	66 (67.3%)	15 (23.1%)	
Concomitant malformations	False	1114 (89.8%)	444 (83.8%)	159 (82.8%)	27 (84.4%)	85 (86.7%)	42 (64.6%)	<0.001 ^1^
	True	126 (10.2%)	86 (16.2%)	33 (17.2%)	5 (15.6%)	13 (13.3%)	23 (35.4%)	

Abbreviations: BV-S: biventricular simple; BV-C: biventricular complex; UVH: univentricular heart disease; ECMO: extracorporeal membrane oxygenation; ICU: intensive care unit. Notes: *p*-values were calculated using one of the following: ^1^ Fisher’s exact test for categorical variables, ^2^ Pearson’s chi-squared test, or ^3^ the Kruskal–Wallis rank sum test. *p*-values indicate the association between each variable and the respective CHD group based on group comparisons for categorical variables and differences in central tendency for continuous variables.

**Table 2:** Final Cox regression model using in-hospital mortality as the dependent variable.

Predictors	Hazard Ratio	CI	*p*
BV-C	2.50	0.99–6.28	0.073
UVH-Ia	4.31 *	1.44–12.90	0.025
UVH-Ib	6.09 *	1.82–20.39	0.010
UVH-IIa	2.44	0.72–8.25	0.203
UVH-IIb	2.96	0.50–17.62	0.313
Age at surgery (days)	0.99	0.99–1.00	0.136
Creatinine ratio	1.74 *	1.19–2.55	0.016
Urea ratio	0.84	0.67–1.05	0.185
Bypass time group [>90 min]	0.90	0.23–3.51	0.889
Leukocytes below 4000/µL after surgery	0.90	0.43–1.86	0.817
Open thorax	0.57	0.29–1.15	0.196
ECMO	13.97 ***	7.02–27.90	<0.001
Dialysis	1.70	0.91–3.17	0.180
Infection	0.79	0.42–1.46	0.528
Unplanned operations	1.03	0.91–1.17	0.723
Minimal temperature during surgery (group 28–32 °C)	0.98	0.29–3.26	0.974
Minimal temperature during surgery (group < 28 °C)	1.16	0.35–3.80	0.831
Malformations	1.14	0.61–2.14	0.731
Weight below 2500 g	8.05 ***	3.37–19.23	<0.001

Notes: * *p* < 0.05, *** *p* < 0.001. Abbreviations: BV-C: biventricular complex; UVH: univentricular heart disease; ECMO: extracorporeal membrane oxygenation.

## Data Availability

The datasets generated and processed in the present study are not subject to public release due to institutional data transfer policies but are available from the corresponding author on reasonable request.

## Supplementary Materials

The following supporting information can be downloaded at: https://www.mdpi.com/article/10.3390/life16060941/s1, Table S1: Classification cardiac disease groups. In instances where groups are defined by both OPS and ICD codes, the respective codes are combined using a logical “AND” between the two code systems (ICD/OPS) and “OR” within a single code system. OPS: German procedure coding system; ICD: International Classification of Diseases; UVH I: univentricular heart disease 1; UVH II: univentricular heart disease 2; BV-C: biventricular complex heart disease; BV-S: biventricular simple heart disease. Table S2: Cohort characteristics stratified by mortality as the primary outcome. The bivariable associations between potential risk factors and mortality are outlined. Data are presented as n (%), median [interquartile range (IQR)], or mean (standard deviation, SD), as appropriate. Percentages are calculated per row. *p*-values were calculated using the following: ^1^ Pearson’s chi-squared test, ^2^ Fisher’s exact test for categorical variables, or ^3^ the Wilcoxon rank-sum test for continuous variables. IQR: interquartile range; SD: standard deviation; ECMO: extracorporeal membrane oxygenation; ICU: intensive care unit. Table S3: Group definitions. The identification of distinct groups is facilitated by the utilization of both OPS and ICD codes, which are then processed through a logical “OR” operation. OPS: German procedure coding system; ICD-10: International Classification of Diseases; ECMO: extracorporeal membrane oxygenation.
