# Supplementary material for: Quality Assessment in Paediatric Cardiology: Experiences from Leveraging a Clinical Data Warehouse
Source: Life (Basel). 2026 Jun 2;16(6):941. doi: 10.3390/life16060941 (PMC13300982; doi:10.3390/life16060941)
Supplement: Supplementary file 1 [file life-16-00941-s001.zip › life-4328165-Table S1.pdf]

Table S 1: Classification cardiac disease groups

In instances where groups are defined by both OPS and ICD codes, the respective codes are combined using a logical 'AND' between the two code systems (ICD/OPS) and 'OR' within a single code system. OPS: German procedure coding system; ICD: internationial classification of diseases; UVH I: univentricular heart disease 1; UVH II: univentricular heart disease 2; BV-C : biventricular complex heart disease; BV-S : biventricular simple heart disease.

Disease group	Heart defect (subcategory)	OPS codes	ICD codes	Anamnesis
UVH Ia	Norwood-Typ-I (HLHS)	5-359.66, 5-359.67	Q23.4	
	Norwood-Typ-I (with aortic arch stenosis)	5-359.66, 5-359.67	Q25.1, Q25.2, Q25.4	
	Norwood-Typ-I (without aortic arch stenosis)	5-359.66, 5-359.67		
UVH Ib	Univentricular heart	5-390	Q20.4, Q22.4, Q23.4, Q25.2	None of these OPS: 5-359.60, 5-359.61, 5-359.62, 5-359.63, 5-359.64, 5-359.65, 5-359.66, 5-359.67
UVH IIa	Glenn + Norwood in anamnesis	5-359.60, 5-359.61, 5-359.62		OPS: 5-359.66, 5-359.67
	Glenn + Palliation with Shunt/Banding in anamnesis	5-359.60, 5-359.61, 5-359.62		OPS: 5-390
	Glenn without surgery	5-359.60, 5-359.61, 5-359.62		
	Fontan + Norwood in anamnesis	5-359.63, 5-359.64, 5-359.65		OPS: 5-359.66, 5-359.67
	Fontan + Shunt in anamnesis	5-359.63, 5-359.64, 5-359.65		OPS: 5-390
	Other correction	5-359.6x		
	Fontan (Glenn)	5-359.63, 5-359.64, 5-359.65		OPS: 5-359.60, 5-359.61, 5-359.62
	Fontan without recorded surgery	5-359.63, 5-359.64, 5-359.65		

UVH IIb	Glenn and/or Fontan with other surgery	5-35, 5-36, 5-37, 5-38, 5-39		OPS: 5-359.60, 5-359.61, 5-359.62, 5-359.63, 5-359.64, 5-359.65
BV-C	Complex biventricular	5-356.6		
	Complex biventricular	(5-359.0) AND (5-356.7)	Q21.2	
	Complex biventricular	(5-359.0) AND (5-356.8)	Q21.2	
	Complex biventricular	5-351.0c, 5-351.0e, 5-357.7, 5-357.8, 5-358.07, 5-358.0e, 5-359.1, 5-359.20, 5-359.4, 5-359.5, 5-359.8, 5-359.x, 5-390		
	Complex biventricular	5-350, 5-351, 5-352, 5-353, 5-354, 5-355, 5-356, 5-357.0, 5-357.1, 5-357.2, 5-357.3, 5-357.4, 5-357.5, 5-357.6, 5-357.9, 5-357.x, 5-357.y, 5-358, 5-359.0, 5-359.21, 5-359.3, 5-359.7	Q20.0, Q20.3, Q20.5, Q20.6, Q20.8, Q22.0, Q22.5, Q22.6, Q23.8, Q25.2, Q25.5	
BV-S	Simple biventricular	5-350, 5-351, 5-352, 5-353, 5-354, 5-355, 5-356, 5-357.0, 5-357.1, 5-357.2, 5-357.3, 5-357.4, 5-357.5, 5-357.6, 5-357.9, 5-357.x, 5-357.y, 5-358, 5-359.0, 5-359.21, 5-359.3, 5-359.7		
