# Supplementary material for: Quality Assessment in Paediatric Cardiology: Experiences from Leveraging a Clinical Data Warehouse
Source: Life (Basel). 2026 Jun 2;16(6):941. doi: 10.3390/life16060941 (PMC13300982; doi:10.3390/life16060941)
Supplement: Supplementary file 1 [file life-16-00941-s001.zip › life-4328165-Table S2.pdf]

Table S2: Cohort characteristics stratified by mortality as the primary outcome.

Variable	Category	Deceased		Total	*p value*
		**FALSE**	**TRUE**		
Sex	M	1192 (96.1%)	48 (3.9%)	1240	0.381 [1]
	W	888 (96.8%)	29 (3.2%)	917	
Age at surgery (days)	Min / Max	0.6 / 6527.4	0.4 / 3575.4	0.4 / 6527.4	< 0.001 [3]
	Med [IQR]	188.4 [73.1;1122.6]	13.4 [6.4;133.4]	181.6 [59.6;1089.4]	
	Mean (std)	942.4 (1535.2)	252.9 (586.7)	917.8 (1517.0)	
Age cohort	Neonates (0-28d)	408 (90.5%)	43 (9.5%)	451	< 0.001 [1]
	Infants (29-365d)	860 (97.5%)	22 (2.5%)	882	
	Small children (1-3 years)	388 (98.2%)	7 (1.8%)	395	
	>= 4 years	424 (98.8%)	5 (1.2%)	429	
Weight (kg)	Min / Max	0.4 / 135.0	1.6 / 38.0	0.8 / 135.0	< 0.001 [3]
	Med [IQR]	6.5 [4.0;13.0]	3.3 [2.9;5.0]	6.3 [4.0;13.0]	
	Mean (std)	12.2 (14.8)	5.4 (5.4)	12.0 (14.6)	

Heart defect	Biventricular simple	1233 (99.4%)	7 (0.6%)	1240	< 0.001 [2]
	Biventricular complex	502 (94.7%)	28 (5.3%)	530	
	Univentricular - Group Ia	79 (80.6%)	19 (19.4%)	98	
	Univentricular - Group Ib	53 (81.5%)	12 (18.5%)	65	
	Univentricular - Group IIa	184 (95.8%)	8 (4.2%)	192	
	Univentricular - Group IIb	29 (90.6%)	3 (9.4%)	32	
Bypass time (min)	Min / Max	0 / 550	0 / 482	0 / 550	< 0.001 [3]
	Med [IQR]	119.0 [65.0;185.2]	193.0 [91.0;253.0]	121.0 [66.0;187.0]	
	Mean (std)	128.1 (87.7)	184.9 (118.0)	130.1 (89.6)	
Minimal temperature during surgery (group)	> 32 °C	631 (96.9%)	20 (3.1%)	651	< 0.001 [1]
	28 - 32 °C	759 (98.1%)	15 (1.9%)	774	
	< 28 °C	690 (94.3%)	42 (5.7%)	732	
Weight below 2500g	False	2025 (96.8%)	67 (3.2%)	2092	< 0.001 [2]
	True	55 (84.6%)	10 (15.4%)	65	
ECMO	False	2016 (98.5%)	30 (1.5%)	2046	< 0.001 [2]
	True	64 (57.7%)	47 (42.3%)	111	

Days on ventilator	Min / Max	0.1 / 217.1	0.04 / 160.0	0.1/ 217.1	< 0.001 [3]
	Med [IQR]	1.0 [0.3;3.9]	16.4 [7.3;37.5]	1.1 [0.3;4.4]	
	Mean (std)	5.5 (16.8)	27.8 (29.1)	6.4 (17.9)	
Days in ICU	Min / Max	0.8 / 169.2	0.2 / 99.2	0.2 / 169.2	< 0.001 [3]
	Med [IQR]	2.2 [1.2;7.0]	15.5 [10.3;44.2]	2.8 [1.2;8.2]	
	Mean (std)	7.5 (15.9)	28.8 (28.5)	8.6 (17.3)	
Dialysis	False	1982 (98.1%)	39 (1.9%)	2021	< 0.001 [2]
	True	98 (72.1%)	38 (27.9%)	136	
Chromosomal abnormalities	False	1807 (96.4%)	68 (3.6%)	1875	0.713 [1]
	True	273 (96.8%)	9 (3.2%)	282	
Infection	False	1817 (97.8%)	41 (2.2%)	1858	< 0.001 [1]
	True	263 (88.0%)	36 (12.0%)	299	
Unplanned operations	False	2024 (97.1%)	60 (2.9%)	2084	< 0.001 [2]
	True	56 (76.7%)	17 (23.3%)	73	
Creatinine change after surgery (%)	Min / Max	0.4 / 8.0	0.7 / 6.0	0.4 / 8.0	< 0.001 [3]
	Med [IQR]	1.2 [1.1;1.4]	1.6 [1.1;2.0]	1.2 [1.1;1.4]	
	Mean (std)	1.3 (0.5)	1.7 (0.9)	1.3 (0.5)	

Urea change after surgery (%)	Min / Max	0.3 / 17.3	0.6 / 8.2	0.3 / 17.3	< 0.001 [3]
	Med [IQR]	1.3 [1.0;1.9]	2.1 [1.2;2.6]	1.3 [1.0;2.0]	
	Mean (std)	1.7 (1.2)	2.4 (1.6)	1.7 (1.2)	
Bypass time group (min)	0 min.	286 (96.6%)	10 (3.4%)	296	0.793 [1]
	>0 - <90 min.	177 (97.3%)	5 (2.7%)	182	
	>= 90 min.	1617 (96.3%)	62 (3.7%)	1679	
Leukocytes below 4,000 after surgery	False	2016 (97.1%)	60 (2.9%)	2076	< 0.001 [2]
	True	64 (79.0%)	17 (21.0%)	81	
Open thorax	False	1939 (98.2%)	36 (1.8%)	1975	< 0.001 [1]
	True	141 (77.5%)	41 (22.5%)	182	
Number of unplanned operations	Min / Max	0 / 14.0	0 / 13.0	0 / 14.0	< 0.001 [3]
	Mean (std)	0.1 (0.7)	0.9 (2.3)	0.1 (0.8)	
Concomitant malformations	False	1823 (97.4%)	48 (2.6%)	1871	< 0.001 [1]
	True	257 (89.9%)	29 (10.1%)	286	

Notes: Data are presented as n (%), median [interquartile range (IQR)], or mean (standard deviation, SD), as appropriate. Percentages are calculated per row. P-values were calculated using [1]: Pearson's chi-squared test or [2]: Fisher's exact test for categorical variables or [3]: the Wilcoxon rank-sum test for continuous variables.

Abbreviations: IQR: interquartile range; SD: standard deviation; ECMO: extracorporeal membrane oxygenation; ICU: intensive care unit
