# Supplementary material for: Quality Assessment in Paediatric Cardiology: Experiences from Leveraging a Clinical Data Warehouse
Source: Life (Basel). 2026 Jun 2;16(6):941. doi: 10.3390/life16060941 (PMC13300982; doi:10.3390/life16060941)
Supplement: Supplementary file 1 [file life-16-00941-s001.zip › life-4328165-Table S3.pdf]

Table S3: Group definitions. The identification of distinct groups is facilitated by the utilization of both OPS and ICD codes, which are then processed through a logical 'OR' operation.

OPS: German procedure coding system; ICD-10: international classification of diseases; ECMO: extracorporeal membrane oxygenation

Group	OPS codes	ICD codes
Concomitant malformations	-	D80-D89, Q00-Q07, Q30-Q39, Q40-Q45, Q50-Q52, Q60-Q64, Q77-Q79, Q80-Q89
Chromosomal abnormalities	-	Q90-99
Pulmonary arterial hypertension	-	I27.00-02, I27.08, I27.20-22, I27.28, I27.8, I27.9
Dialysis	8-853, 8-854, 8-855, 8-857	-
ECMO	5-376, 8-852.0, 8-852.2, 8-852.3, 8-852.4, 8-852.5, 8-852.6	-
Cardiac arrest	8-771	I46
Open thorax	5-916.7a	M96.80
Heart/lung transplantation	5-375	-
Infection	-	P23, P36, P39.3, P39.8, P39.9, P77, P78.0, P78.1, J10-18, J20-22, J85-86, J98.5, J98.7, J98.8, J98.9, T81.4, I30, K63, K65, K61
